# Scalable Production of Transfection-Grade Plasmid DNA by Liquid–Liquid Extraction Without Chromatography: Application to CAR-T Vector Packaging

**DOI:** 10.3390/bioengineering13080939

**Published:** 2026-08-20

**Authors:** Gaziza Nigmatulla, Aitolkyn Kydyrbayeva, Tolganay Kulatay, Gulzat Zauatbayeva, Bakytkali Ingirbay, Lyazzat Sagyndykova, Viktoriya Keyer, Dinara Zharlyganova, Maral Zhumabekova, Alexandr V. Shustov

**Affiliations:** National Center for Biotechnology, Korgalzhin hwy 3/5, 010000 Astana, Kazakhstan; nigmatullag@gmail.com (G.N.); aitolkyn.yk@gmail.com (A.K.); kulatay@biocenter.kz (T.K.); zauatbaeva@biocenter.kz (G.Z.); ingirbay@biocenter.kz (B.I.); sagyndykova@biocenter.kz (L.S.); keer@biocenter.kz (V.K.); dinarazh@mail.ru (D.Z.); zhumabekova@biocenter.kz (M.Z.)

**Keywords:** plasmid DNA purification, acidic phenol extraction, lentiviral vector production, CAR-T-cell therapy, transfection-grade plasmid, cost-effective manufacturing, gene therapy

## Abstract

The growing demand for high-quality plasmid DNA (pDNA) in cell and gene therapy, including chimeric antigen receptor T-cell therapy (CAR-T) manufacturing, is constrained by the limited availability of transfection-grade plasmids. This study describes a practical, scalable, and cost-effective acidic phenol extraction method based on a modified alkaline lysis protocol. This approach efficiently removes endotoxins and residual genomic DNA without requiring chromatography or ultracentrifugation. When benchmarked against CsCl density gradient ultracentrifugation and a commercial midiprep kit, the acidic phenol method delivered comparable plasmid purity and functional performance while providing superior scalability, yielding 5–10 mg of pDNA per 500 mL culture. Notably, residual endotoxin levels were negligible for downstream applications such as transfection and lentiviral vector packaging. Lentiviral vectors produced with these plasmids reached titers exceeding 5 × 10^6^ TU/mL, with transduction efficiencies statistically indistinguishable from those obtained with CsCl-purified DNA. The acidic phenol extraction method is a robust, scalable, and cost-effective alternative to industry-standard methods, matching them in yield and purity while being readily scalable, making it a practical tool for both academic research and preparative laboratory production.

## 1. Introduction

The rise of gene therapy, nucleic acid vaccines, and the application of genetically modified cells for therapeutic purposes—exemplified by chimeric antigen receptor T-cell (CAR-T) therapy—has generated a critical demand for the large-scale supply of high-quality plasmid DNA (pDNA) derived from Escherichia coli [1,2]. These plasmids serve directly as gene delivery vehicles in DNA vaccination and RNA vaccine production and, under current dominant technologies, as indispensable intermediate materials for producing viral vectors that are widely employed to deliver therapeutic genes in cell and gene therapy (CGT) [3]. Lentiviruses (LVs) and adeno-associated viruses (AAVs), the primary delivery vehicles for these advanced therapies, depend on high-quality pDNA for their production [3].

LV production is most commonly achieved both in industry and academia using transient plasmid transfection of suitable packaging cell cultures with a mixture of plasmid DNAs containing the transfer vector, which encodes the viral genome, and helper plasmids that express the various components of the packaging system [4,5]. Plasmid transfection is currently the preferred method because it offers flexibility, speed, and simplicity: different plasmid combinations can be readily adjusted to produce diverse vector constructs without the need to generate stable producer cell lines, which are time-consuming and labor-intensive to establish. This transient approach is particularly advantageous for research settings and academic CAR-T manufacturing, where multiple vector designs may need to be tested and optimized.

Regulatory guidelines mandate stringent purity criteria for plasmid DNA intended for therapeutic use, requiring pDNA to be predominantly in the covalently closed circular form (cccDNA), with minimal contamination by RNA, protein, residual bacterial genomic DNA, and endotoxin [6,7]. Endotoxin (lipopolysaccharide from the outer membrane of Gram-negative bacteria) can trigger severe inflammatory responses and reduce transfection efficiency, making its removal a significant task during purification of plasmids intended for therapeutic applications. For clinical applications, acceptable levels are often below 0.1 endotoxin units (EU) per µg of pDNA [8].

Laboratory-scale plasmid DNA purifications are frequently performed using commercial kits, which offer end-user convenience, simplicity, and speed. However, these kits are typically expensive and have limited scalability, restricting their application in industrial settings [9]. Another well-established approach for obtaining highly pure plasmid DNA, predominantly in the cccDNA form, is cesium chloride (CsCl) density gradient ultracentrifugation in the presence of ethidium bromide. Long considered the “gold standard” for plasmid purification, this method yields DNA of exceptional purity but requires costly equipment, including an ultracentrifuge and dedicated rotors [10]. Another purification strategy—liquid–liquid extraction—has been used in various protocols to remove impurities such as proteins, host DNA, and endotoxin, typically as a preparatory step before final chromatographic purification [11,12,13].

Industrial-scale pDNA purification relies primarily on chromatographic methods [14,15]. The most common techniques are anion-exchange chromatography (AEC) and hydrophobic interaction chromatography (HIC), followed by size-exclusion chromatography (SEC) as a polishing step. AEC removes proteins, low-molecular-weight RNA, and endotoxins, while HIC separates supercoiled pDNA from open circular isoforms and more hydrophobic impurities, including endotoxins and denatured DNA. More recently, thiophilic aromatic adsorption chromatography (e.g., Cytiva’s PlasmidSelect Xtra) has emerged for purifying supercoiled covalently closed circular (ccc) pDNA. For example, Urthaler et al. [3] describe a process using three chromatographic steps (HIC, AEC, and SEC) followed by tangential flow filtration (TFF) for concentration. Such multi-step processes yield pDNA with up to 98% cccDNA, undetectable RNA, a protein content below 1 µg/mg pDNA, and endotoxin levels below 0.1 EU/mg pDNA. Novel approaches, such as multimodal chromatography, aim to simplify the production line [16]. The purification step remains the costliest operation during pDNA production, accounting for up to 80% of total manufacturing costs [14].

In the present study, we describe a liquid–liquid extraction method that yields high-quality pDNA preparations with milligram-scale recovery, without the need for commercial kits or additional orthogonal purification steps such as chromatography or CsCl gradient ultracentrifugation.

## 2. Materials and Methods

### 2.1. Bacterial Strain, Cells and Media

*E. coli* DH5α strain was used for plasmid propagation and large scale plasmid isolation. Bacterial cells were grown in Terrific Broth (TB) medium (Sigma Aldrich, St. Louis, MO, USA, Cat. T0918).

HEK293FT cells (Thermo Fisher Scientific, Waltham, MA, USA, Cat. R70007) were used for lentiviral vector packaging and functional titer determination. Cells were cultured in complete DMEM (high glucose; Gibco, Grand Island, NY, USA, Cat. 11965092) supplemented with 10% fetal bovine serum (FBS; Gibco, Grand Island, NY, USA, Cat. 10091148), 1% penicillin–streptomycin, 1% non-essential amino acids (NEAAs), and 2 mM L glutamine (all from Gibco, Grand Island, NY, USA). Cells were maintained at 37 °C in a 5% CO_2_ atmosphere and passaged upon reaching 90% confluence.

### 2.2. Plasmids

The lentiviral transfer vector LV/CAR-GFP encodes a chimeric antigen receptor (CAR) targeting the human cell surface marker CD19. The CAR is fused at its cytoplasmic tail to GFP via an uncleavable linker, as described previously [17]. For LV packaging, we used the second-generation packaging system, comprising psPAX2 (Addgene, Watertown, MA, USA, Cat. 12260), which expresses HIV-1 capsid and enzymatic proteins, and the VSV-G envelope-expressing plasmid pMD2.G (Addgene, Watertown, MA, USA, Cat. 12259).

### 2.3. Biomass Production for Plasmid DNA Isolation

Plasmids were propagated in E. coli DH5α cells in Terrific Broth (TB) medium (Sigma Aldrich, St. Louis, MO, USA, Cat. T0918) supplemented with appropriate antibiotics. Starter cultures (5 mL) were grown at 37 °C for 8–10 h with shaking at 150 rpm. For preliminary experiments, 50 mL cultures in 200 mL flasks were used for all three purification methods. To evaluate scalability, acidic phenol and CsCl gradient methods were then scaled up to 500 mL cultures in 2 L flasks. Cells from multiple culture batches were pooled, harvested by centrifugation (6000 rpm, 10 min, 4 °C), and pellets of equal weight were frozen for subsequent purification. For the commercial midiprep kit, due to its limited binding capacity, the culture volume remained at 50 mL as recommended by the manufacturer. For the preparative-scale experiments (500 mL cultures), three independent biomass production batches were prepared on different days, and each batch was processed separately through the subsequent purification methods. Plasmids were purified as described in Section 2.4, Section 2.5 and Section 2.7.

### 2.4. Plasmid DNA Isolation by CsCl Density Gradient Ultracentrifugation

For plasmid isolation from small-scale cultures (50 mL, Section 2.3), the bacterial pellet was resuspended in 2 mL of buffer BF1 (0.025 M Tris HCl, 0.1 M NaCl, 0.01 M EDTA, pH 7.5). Then 4 mL of buffer BF2 (0.2 M NaOH, 1% SDS) was added, and the mixture was mixed until clear and homogeneous, indicating cell lysis. The lysate was incubated at room temperature for 5 min. Next, 3 mL of ice-cold buffer BF3 (3 M potassium acetate, pH 5.5) was added, mixed, and incubated on ice for 10 min. Insoluble debris was pelleted by centrifugation at 6000 rpm (A-4-81 rotor, Eppendorf 5810R centrifuge; Eppendorf, Hamburg, Germany) for 10 min at 4 °C. The supernatant was transferred to a 15 mL tube, avoiding the pellet. An equal volume of isopropanol was added, mixed, and kept at −20 °C for 15 min to precipitate nucleic acids. The precipitate was collected by centrifugation at 4000 rpm (same rotor and centrifuge) for 10 min at 4 °C. The supernatant was discarded, and the pellet was dissolved in 1 mL of TE buffer (10 mM Tris HCl, 1 mM EDTA, pH 7.5). One ml of 5 M lithium chloride (LiCl) solution in water was added, vortexed, and left on ice for 30 min to precipitate high-molecular-weight RNA. The RNA was removed by centrifugation at 4000 rpm for 10 min at 4 °C (same rotor and centrifuge). DNA was again precipitated with an equal volume of isopropanol. After pelleting, the supernatant was removed, and the pellet was dissolved in 1 mL of TE buffer. This plasmid preparation was used for further purification by cesium chloride (CsCl) density gradient ultracentrifugation, as described below.

For preparative-scale purification of plasmid DNA (from 500 mL bacterial cultures, Section 2.3), the bacterial pellet was resuspended in buffer BF1 to approximately 30% (w/v), and the volume was adjusted to 10 mL with the same buffer. Then, 20 mL of buffer BF2 was added, and the mixture was mixed with a glass rod until the lysate became clear and homogeneous. The lysate was incubated at room temperature for 10 min. Next, 15 mL of ice-cold buffer BF3 was added, mixed, and incubated on ice for 15 min. Insoluble debris was pelleted by centrifugation at 6000 rpm (JA-10 rotor, Avanti J26S XP centrifuge, Beckman Coulter) for 10 min at 4 °C. The supernatant was transferred to 50 mL tubes, avoiding the pellet. An equal volume of isopropanol was added, mixed, and kept at −20 °C for 15 min to precipitate nucleic acids. The precipitate was collected by centrifugation at 4000 rpm (A-4-81 rotor, Eppendorf 5810R centrifuge; Eppendorf, Hamburg, Germany) for 10 min at 4 °C. The supernatant was discarded, and the pellet was dissolved in 2 mL of TE buffer. An equal volume of 5 M lithium chloride (LiCl) solution in water was added, vortexed, and left on ice for 30 min followed by centrifugation at 4000 rpm for 10 min at 4 °C (same rotor and centrifuge). DNA was again precipitated with an equal volume of isopropanol. After pelleting, the supernatant was removed, and the pellet was dissolved in 1 mL of TE buffer. This crude plasmid preparation was used for purification by banding in CsCl gradients.

To prepare a gradient for ultracentrifugation, 4.3 g of dry CsCl (Sigma Aldrich, St. Louis, MO, USA, Cat. C4036) was weighed in a tared 15 mL tube, then 1 mL of TE buffer was added, followed by the plasmid DNA solution (1 mL in TE) and 40 μL of ethidium bromide solution (10 mg/mL; Sigma Aldrich, St. Louis, MO, USA, Cat. E7637). TE buffer was added to bring the total weight of the mixture to 8.1 g and the CsCl was dissolved completely by mixing. The solution was transferred to 4.9 mL OptiSeal tubes (Beckman Coulter, Brea, CA, USA, Cat. 362185). The tubes were capped according to the manufacturer’s instructions, placed in an NVT90 rotor (Beckman Coulter, Brea, CA, USA), and centrifuged at 65,000 rpm for 14–18 h at 22 °C using an Optima XPN-90 ultracentrifuge (Beckman Coulter, Brea, CA, USA). After centrifugation, the lower band (cccDNA) was collected by puncturing the tube with a needle and aspirating the corresponding portion of the gradient. The plasmid solution was transferred to a 15 mL tube, mixed with 1 mL of TE buffer and 4.5 mL of 96% ethanol, and kept at −20 °C for 15 min to precipitate the DNA. The DNA was pelleted by centrifugation at 4000 rpm (A-4-81 rotor, Eppendorf 5810R centrifuge) for 10 min at 4 °C. The supernatant was discarded, and the pellet was dissolved in 0.4 mL of TE buffer and transferred to a 2 mL microcentrifuge tube. To remove ethidium bromide, 0.4 mL of phenol–chloroform (1:1 mixture; Sigma Aldrich, St. Louis, MO, USA, Cat. 77617) was added, vortexed vigorously, and centrifuged for 10 min at 10,000 rpm. The upper aqueous phase was transferred to a fresh microcentrifuge tube. Then, 0.1 volume of 5 M NaCl and an equal volume of isopropanol were added, mixed, and kept at −20 °C for 15 min. The DNA was pelleted by centrifugation. The pellet was washed with 0.5 mL of 70% ethanol, followed by removal of the liquid and air drying. Finally, the purified plasmid DNA pellet was dissolved in TE buffer. All preparative-scale CsCl gradient purifications were performed in three biological replicates (three separate bacterial pellets, each processed on a different day).

DNA concentration and purity were assessed by measuring absorbance at 260 nm (A_260_) and the A_260_/A_280_ ratio, then the concentration was adjusted to 1 mg/mL. Plasmid integrity and topology were verified by agarose gel electrophoresis of undigested DNA and by restriction analysis with selected endonucleases.

### 2.5. Plasmid DNA Isolation Using Endotoxin-Free Midi Kit

Plasmids were isolated using the QIAfilter Plasmid Midi Kit (Qiagen, Hilden, Germany, Cat. No. 12243) and the EndoFree Plasmid Buffer Set (Qiagen, Cat. No. 19048). Bacterial cell pellets from 50 mL cultures were resuspended in 4 mL of buffer P1 (supplemented with 100 µg/mL RNase A). Bacterial cells were lysed by adding 4 mL of buffer P2 and incubating for 5 min. Then, 4 mL of buffer P3 was added, and the mixture was poured into the QIAfilter Midi Cartridge. After a 10 min incubation at room temperature the lysate was allowed to pass through the cartridge and collected. Buffer ER was added to the collected lysate at 0.1 volumes and incubated on ice for 30 min. A column (QIAGEN-tip 100) was equilibrated with Buffer QBT. The cleared lysate was passed through the QIAGEN-tip, which was then washed twice with 10 mL of buffer QC. DNA was eluted with 5 mL of buffer QN. Plasmid DNA was precipitated with isopropanol. The DNA pellet was washed with 2 mL of 70% ethanol. After air-drying until no visible liquid remained, the DNA was dissolved in TE. DNA concentration and purity were assessed as described in Section 2.4.

### 2.6. Preparation of Buffered Phenol (pH 5.5)

Crystalline phenol (Sigma-Aldrich, St. Louis, MO, USA, Cat. 16016) was redistilled using a laboratory distillation apparatus. The colorless phenol crystals were mixed with an equal volume of MQ water. The mixture was allowed to stand until complete phase separation occurred, upon which the upper aqueous phase was removed. An equal volume of equilibration buffer (50 mM sodium acetate, 75 mM NaCl, pH 5.5) was added to the liquid water-saturated phenol, and the mixture was shaken to ensure thorough mixing. The mixture was again allowed to stand for phase separation. The aqueous phase was then removed, and its pH was measured. This equilibration step was repeated with fresh buffer until the pH of the aqueous phase reached 5.5 ± 0.1. The resulting buffered phenol was aliquoted into sterile tubes, and stored at −20 °C until use.

### 2.7. Plasmid DNA Isolation by Modified Alkaline Lysis Followed by Acidic Phenol Extraction

For plasmid isolation from small-scale cultures (50 mL, Section 2.3), the bacterial pellet was resuspended in 2 mL of buffer E (40 mM Tris acetate, 2 mM EDTA, pH 7.9). Then, 4 mL of alkaline SDS buffer (3% SDS, 50 mM Tris, pH 12.6) was added, and the mixture was mixed until homogeneous. The tube was placed in a water bath at 55 °C for 1 h to denature chromosomal DNA. After cooling on ice for 5 min, 3 mL of ice-cold 3 M potassium acetate (equilibrated with acetic acid to pH 5.5) was added, and incubated on ice for 10 min to precipitate SDS–protein complexes and denatured chromosomal DNA. Subsequently, 0.5 mL of chloroform was added, the mixture was vigorously shaken, and centrifuged at 6000 rpm (A-4-81 rotor, Eppendorf 5810R centrifuge; Eppendorf, Hamburg, Germany) for 10 min at 4 °C. The aqueous phase was collected, and nucleic acids were precipitated with an equal volume of isopropanol upon incubation at −20 °C for 15 min. The crude DNA pellet was obtained by centrifugation (4000 rpm, 10 min, 4 °C), dissolved in 1 mL of TE buffer, treated with an equal volume of 5 M LiCl on ice for 30 min to precipitate high-molecular-weight RNA, and centrifuged (4000 rpm, 15 min, 4 °C). The supernatant containing plasmid DNA was transferred to a new tube, precipitated with isopropanol in the presence of 0.1 volume of 5 M NaCl, and the pellet was dissolved in 0.5 mL of TE buffer. To degrade residual RNA, RNase A (Sigma-Aldrich, St. Louis, MO, USA, Cat. No. R4875) was added to a final concentration of 10 μg/mL, and the mixture was incubated at 37 °C for 1 h.

To remove endotoxin and residual fragments of linear genomic DNA or relaxed (nicked) plasmid DNA, the plasmid solution after RNase treatment was first precipitated with 0.1 volume of 5 M NaCl and an equal volume of isopropanol. The resulting pellet was dissolved in 500 μL of acidic extraction buffer (50 mM sodium acetate, 75 mM NaCl, pH 4.0). The same volume of phenol (equilibrated to pH 5.5) was added to the DNA solution. The mixture was vortexed vigorously for at least 1 min to ensure the formation of a fine emulsion, then centrifuged at 10,000× *g* for 10 min at room temperature. After centrifugation, three distinct phases formed: a lower organic (phenol) phase, a white interphase layer containing endotoxin, denatured proteins, and extracted linear or nicked DNA, and an upper aqueous phase containing supercoiled plasmid DNA.

The upper aqueous phase was transferred to a new tube, avoiding the interphase. The described procedure of acidic phenol extraction was repeated two more times. A final extraction was performed using 500 μL of chloroform. Plasmid DNA was then precipitated as described. The resulting pellet was washed with 100 μL of 70% ethanol, air-dried, and dissolved in TE buffer.

For preparative-scale purification of plasmid DNA from 500 mL bacterial cultures (Section 2.3), the bacterial pellet was resuspended in 10 mL of buffer E. Then, 20 mL of the alkaline SDS buffer was added, and the mixture was mixed until homogeneous. The tube was placed in a water bath at 55 °C for 1 h. After cooling on ice for 5 min, 15 mL of ice-cold 3 M potassium acetate pH 5.5 was added, and incubated on ice for 30 min. Then, 5 mL of chloroform was added, the mixture was vigorously shaken, and centrifuged at 6000 rpm (JA-10 rotor, Avanti J26S XP centrifuge, Beckman Coulter) for 10 min at 4 °C. The aqueous phase was collected, and nucleic acids were precipitated with an equal volume of isopropanol. The crude DNA pellet was dissolved in 2 mL of TE buffer, treated with an equal volume of 5 M LiCl on ice for 30 min to precipitate ribosomal RNAs, and centrifuged (4000× *g*, 15 min, 4 °C). The supernatant was transferred to a new tube, precipitated with an equal volume of isopropanol in the presence of 0.5 M NaCl, and the pellet was dissolved in 1 mL of TE buffer. Residual RNA was degraded by treatment with RNase A (10 μg/mL) 37 °C for 1 h. The plasmid solution was again precipitated with isopropanol as described.

Acidic phenol extraction of impurities from scaled pDNA preparations was done as follows. The crude pDNA pellet was dissolved in 2 mL of the acidic extraction buffer (50 mM sodium acetate, 75 mM NaCl, pH 4.0) and the solution was distributed by 1 mL portions into 2 mL microcentrifuge tubes. One milliliter of phenol (equilibrated to pH 5.5) was added to each tube. The tubes were vortexed vigorously for at least 1 min to ensure complete mixing and the formation of a fine emulsion, then centrifuged at 10,000× *g* for 10 min at room temperature. The aqueous phase was transferred to a new tube, avoiding the interphase. The described procedure of acidic phenol extraction was repeated two more times. A final extraction was performed using 500 μL of chloroform per tube. Plasmid DNA was then precipitated. The resulting pellets were washed with 200 μL of 70% ethanol, air-dried, and dissolved in TE buffer. Solutions containing the same plasmid from different tubes were pooled, and plasmid purity and integrity were verified as described in Section 2.4. Preparative-scale acidic phenol extractions were carried out in three biological replicates (three pellets from 500 mL cultures were processed on separate days).

### 2.8. Analysis of Organic Phase Extracts and Purified Plasmid Preparations

To monitor removal of nucleic acid contaminants, the organic phases from the first, second, and third acidic phenol extractions, and from the chloroform extraction (Section 2.7), were retained. Each organic phase was mixed with an equal volume of TE buffer, vortexed, adjusted to pH 7.0–7.5 with 1 M Tris (pH 12.4), and centrifuged at 10,000× *g* for 10 min at room temperature. The upper aqueous phase was transferred to new tubes and precipitated with 0.1 volume of 5 M NaCl and an equal volume of isopropanol. Pellets were dissolved in TE and analyzed on a 1% agarose gel with ethidium bromide staining.

To detect potential contamination with single-stranded DNA fragments, the final plasmid preparations were analyzed on agarose gels with intentional overloading (5 µg per lane). Gels were stained with SYBR Green I (Invitrogen Cat. S7567, Waltham, MA, USA) and imaged using a GelDoc XR+ system (Bio-Rad Laboratories, Inc., Hercules, CA, USA).

### 2.9. Lentiviral Vector Packaging

HEK293FT cells were seeded in 150 mm dishes (P150) at a density of 1 × 10^5^ cells/cm^2^. For each dish, a total of 10 μg of plasmid DNA (pLV/CAR-GFP, psPAX2, and pMD2.G at a molar ratio of 2:1.5:1) was diluted in 2.5 mL of serum-free DMEM. Separately, 30 μL of a 1 mg/mL polyethylenimine (PEI MAX, 25 kDa linear; Polysciences, Warrington, PA, USA, Cat. 24765 1) stock solution was diluted in 2.5 mL of serum-free DMEM. The diluted polyethylenimine solution was added to the diluted DNA solution and mixed briefly by vortexing. The resulting mixture was incubated for 15–20 min at room temperature (RT) to allow polyplex formation.

Before addition of the transfection mixture, the culture medium was removed, and 5 mL of the polyplex mixture was added immediately to the cell monolayer and distributed evenly. The cells were incubated for 4 h at 37 °C in a 5% CO_2_ atmosphere. Subsequently, 20 mL of complete DMEM was added to each dish without removing the transfection mixture, and the cells were incubated for an additional 48 h. The culture supernatant was then collected, clarified by centrifugation at 300× *g* for 10 min, filtered through a 0.45 μm PES membrane (Pall Corporation, Port Washington, NY, USA, Cat. 515 0157), aliquoted, and stored at −80 °C until use.

For each purification method, LV packaging was performed in three biological replicates. For the preparative-scale acidic phenol and CsCl methods, each of the three independently purified plasmid batches was used for a separate transfection; for the commercial kit, a single plasmid batch obtained with the kit was selected for all three transfections.

### 2.10. Determination of Lentiviral Functional Titer

Lentiviral functional titers (transducing units, TU/mL) were determined by transducing HEK293FT cells with serial dilutions, followed by flow cytometric analysis. HEK293FT cells were seeded in 6-well plates at 4 × 10^5^ cells per well and allowed to attach. Serial dilutions of LV preparations were prepared in complete DMEM containing 8 μg/mL polybrene (Sigma Aldrich, St. Louis, MO, USA, Cat. H9268) and added to the cells. After 12 h, the polybrene-containing medium was replaced with standard complete DMEM. Preliminary experiments used dilutions from 1:10 to 1:10,000 to estimate the titer range. Subsequently, titrations were performed with dilutions from 1:10 to 1:1000 to achieve 1–20% transduced cells in the final well. At 48 h after replacement of polybrene-containing medium, cells were harvested for analysis.

For the LV/CAR-GFP vector, transduction efficiency was assessed by quantifying GFP positive cells. Cells were detached with trypsin EDTA (Gibco, Grand Island, NY, USA), washed with PBS, and resuspended in MACSQuant Running Buffer (RB; Miltenyi Biotec, Bergisch Gladbach, Germany, Cat. 130 092 747). At least 50,000 events were acquired per sample. Gates for positive cells were set using naïve HEK293FT cells as a negative control, with <0.1% events in the positive gate. MACSQuant Calibration Beads (Miltenyi Biotec, Bergisch Gladbach, Germany, Cat. 130 093 607) were used to monitor gate stability across experiments. Data were analyzed using MACSQuantify Software v.2.13 (Miltenyi Biotec, Bergisch Gladbach, Germany).

Functional titers were determined for each of the three independent LV batches per method. The functional titer (TU/mL) was calculated as:

TU/mL = (% positive cells/100) × (number of cells at transduction) × (dilution factor)

### 2.11. p24 ELISA and Calculation of Specific Activity

To assess the quality of lentiviral particles produced with plasmids purified by different methods, we measured the concentration of HIV-1 p24 capsid protein in the clarified supernatants from the transfections described in Section 2.9. The p24 concentration was determined using a Lenti-X p24 Rapid Titer Kit (Takara Bio USA, Mountain View, CA, USA, Cat. 631476) according to the manufacturer’s instructions.

Briefly, serial 10-fold dilutions of LV-containing supernatants were prepared in the diluent buffer provided with the kit. Aliquots (100 μL) of diluted samples were added to anti-p24-coated wells of the ELISA plate, followed by 20 μL of lysis buffer, and incubated for 30 min at room temperature. Then, 100 μL of HRP-conjugated anti-p24 antibody was added and incubated for an additional 30 min. After six washes with wash buffer, 100 μL of 3,3′,5,5′-tetramethylbenzidine (TMB) substrate was added, and the plate was incubated for 30 min in the dark. The reaction was stopped with 100 μL of stop solution, and the optical density was measured at 450 nm. The p24 concentration (ng/mL) was calculated using a standard curve generated from the p24 standard supplied with the kit.

p24 concentration was measured in each of the three independent LV batches per method. The ELISA assay for each batch was performed in technical duplicates. Specific activity was calculated as the ratio of functional titer to p24 concentration in the same sample:

Specific activity (TU/pg p24) = Functional titer (TU/mL)/p24 concentration (pg/mL)

### 2.12. Endotoxin Quantification

Endotoxin levels in plasmid DNA preparations were determined using the ToxinSensor Chromogenic LAL Endotoxin Assay Kit (GenScript, Piscataway, NJ, USA, Cat. No. L00350) according to the manufacturer‘s instructions. Briefly, the lyophilized LAL reagent was reconstituted with 1.7 mL of water provided with the kit. Plasmid DNA samples (100 µL) were mixed with 100 µL of the LAL reagent, and incubated at 37 °C for the time specified on the kit label. Following incubation, 100 µL of chromogenic substrate was added to each vial and incubated for an additional 6 min at 37 °C. The reaction was stopped by sequential addition of 500 µL of color-stabilizer #1 (Stop Solution), then 500 µL of color-stabilizer #2, and 500 µL of color-stabilizer #3. The absorbance was measured at 545 nm. The *E. coli* endotoxin standard supplied with the kit was serially diluted to generate a standard curve spanning 0.1 to 1 EU/mL. Endotoxin concentrations in samples were determined by interpolation within the standard curve and are expressed as EU per µg of plasmid DNA. Endotoxin levels were assessed in plasmid DNA batches obtained from three independent preparative-scale isolations using the manual purification method.

### 2.13. Statistical Analysis

Data are presented as mean ± standard deviation (SD) from three biological replicates. To compare plasmid yields between the acidic phenol and CsCl methods, we performed Two-Way Repeated Measures ANOVA (RM ANOVA), with purification method (acidic phenol vs. CsCl) and plasmid type (pLV/CAR-GFP, psPAX2, pMD2.G) as within-subjects factors, and each plasmid-batch combination as the subject (six subjects: three plasmids × two batches). This design accounts for the fact that different plasmids inherently yield different amounts of DNA, irrespective of the purification method used.

Lentiviral functional titers and specific activities were compared among the three purification methods (acidic phenol, CsCl gradient, and commercial midiprep kit) using one-way ANOVA with Tukey’s post hoc test for multiple comparisons. All statistical analyses were conducted using GraphPad Prism version 9.3.1 (GraphPad Software, San Diego, CA, USA). Significance levels are denoted as: ns (not significant, *p* > 0.05), * (*p* ≤ 0.05), ** (*p* ≤ 0.01), and *** (*p* ≤ 0.001).

## 3. Results

### 3.1. Overview of the Three Purification Methods

**Figure 1:**
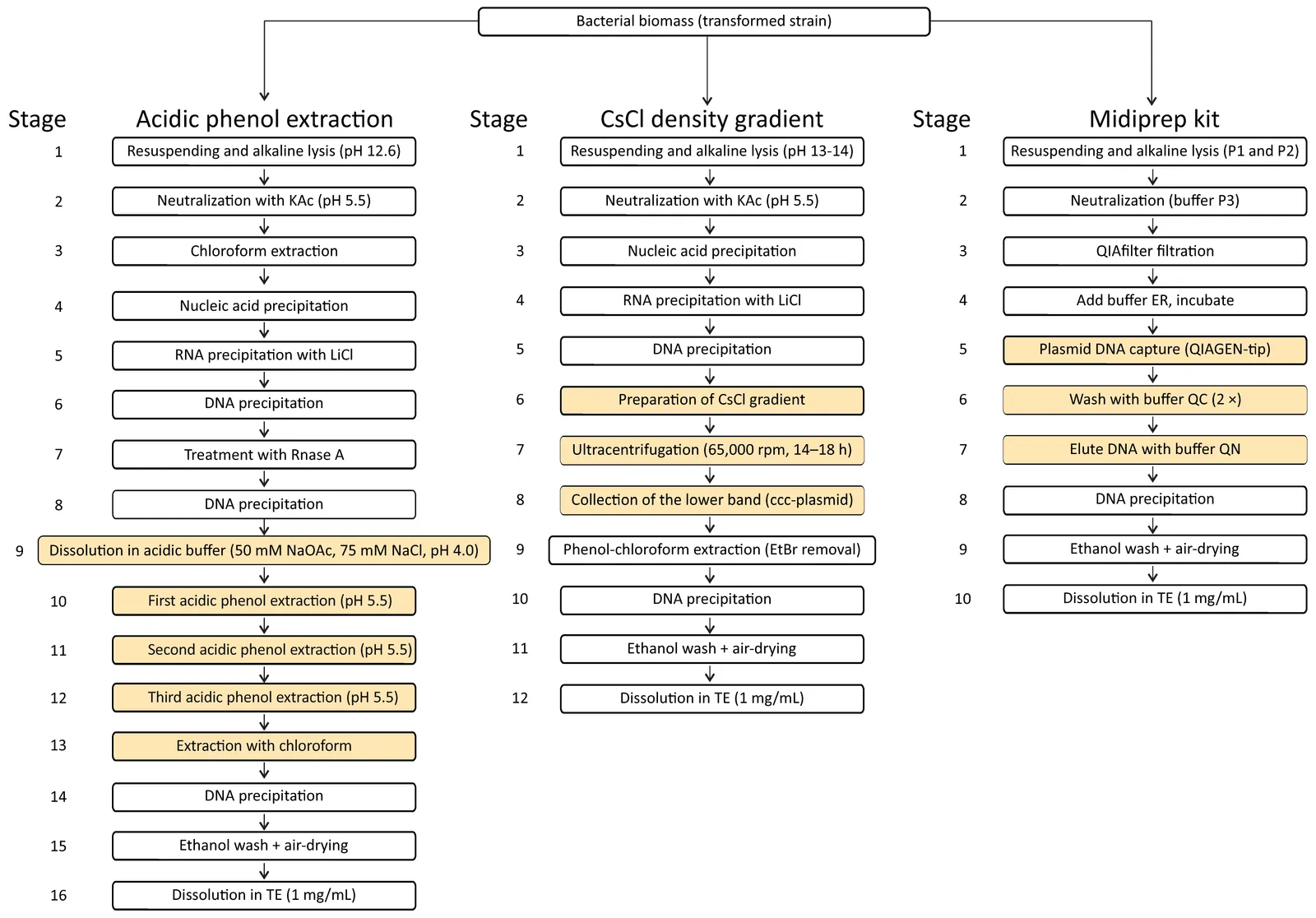
Schematic representation of the three plasmid DNA purification methods. Method with acidic phenol extraction uses only standard equipment and is scalable to preparative amounts (5–10 mg per 500 mL culture). Method with CsCl gradients requires an ultracentrifuge and yields highly pure DNA. The commercial Midi Kit served as a benchmarking control; it removes endotoxin with buffer ER followed by anion-exchange chromatography, but is limited to ~100 µg per column. Yellow highlighting marks the core principle-defining steps that distinguish each protocol.

To compare the performance of the acidic phenol method with the classical CsCl gradient and a widely used commercial kit, plasmid DNA was isolated from parallel bacterial cultures using all three protocols. The experimental workflows are illustrated in Figure 1.

### 3.2. Evaluation of Nucleic Acid Impurity Removal by Acidic Phenol Extraction

**Figure 2:**
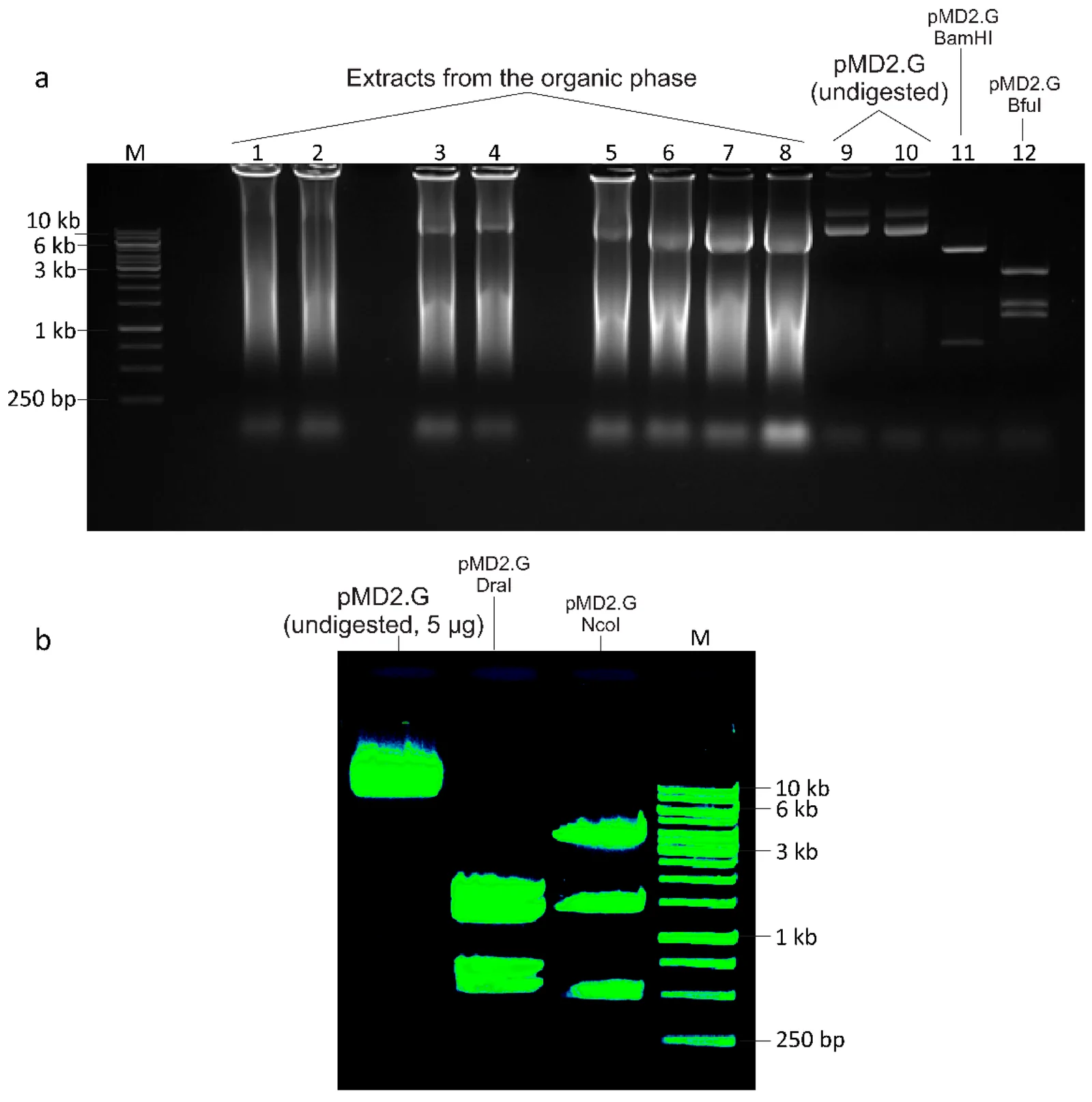
Monitoring of nucleic acid extraction and quality of final plasmid DNA products using the acidic phenol method. (**a**) Analysis of nucleic acid extraction efficiency. Two independently produced bacterial pellets (batch #1 and #2) were processed in parallel. Samples were prepared as described in Section 2.7 and Section 2.8 and run on a 1% agarose gel stained with ethidium bromide. Lanes 1–2: organic phase after the first phenol extraction; lanes 3–4: after the second phenol extraction; lanes 5–6: after the third phenol extraction; lanes 7–8: after chloroform extraction. Lanes 9–10: final pMD2.G plasmid preparations. Lane 11: pMD2.G digested with BamHI (expected fragments: 4964, 827, and 31 bp); lane 12: pMD2.G digested with BfuI (expected fragments: 2779, 1527, 1318, and 198 bp). High-molecular-weight DNA retained in the wells in phenolic extracts (lanes 1–6) confirms efficient removal of genomic DNA. The final undigested plasmid (lanes 9–10) displays a clean supercoiled profile; restriction digests yielded expected fragments without contaminant bands. Lane M: GeneRuler 1 kb DNA ladder (Thermo Fisher Scientific, Waltham, MA, USA, Cat. SM0311). Fragment sizes in kb are shown. (**b**) Assessment of single-stranded DNA contamination. Plasmid preparations were loaded in excess (5 µg per lane) to intentionally overload the lanes and reveal any contaminating single-stranded DNA. Lanes: pMD2.G undigested (5 µg); pMD2.G digested with DraI (expected fragments: 1743, 1406, 1351, 692, and 558 bp); pMD2.G digested with NcoI (expected fragments: 3819, 1487, and 516 bp). The gel was stained with SYBR Green I and photographed using a GelDoc XR+ imager (Bio-Rad Laboratories, Inc., Hercules, CA, USA). The absence of a visible smear confirms that the acidic phenol-purified plasmid preparations are free from significant single-stranded DNA contamination.

To monitor the removal of non-plasmid nucleic acid contaminants extracted into the organic phase during the acidic phenol extraction protocol (Section 2.7), material was back-extracted from the phenol phases obtained at successive stages and analyzed on 1% agarose gels alongside the final purified plasmids. Results from a representative experiment are shown in Figure 2a.

Nucleic acids extracted into the phenol phase contained high-molecular-weight material that remained in the wells (lanes 1–6, Figure 2a). A diffuse signal in the 1–3 kb range increased with successive extractions (from lanes 1–2 to 5–6) and appeared as the dominant contaminant in the chloroform-extracted material (lanes 7–8). This likely represents bacterial chromatin fragments associated with tightly bound proteins. The absence of this material from the final plasmid preparations (lanes 9–10) indicated its efficient removal. The final undigested plasmid preparations (lanes 9–10) displayed a clean supercoiled profile with two distinct bands: a lower, more intense band (~6 kb for pMD2.G, Figure 2a) corresponding to the monomeric supercoiled form and an upper, less intense band representing the non-covalent dimer (catenane) (Figure 2a). Electrophoresis confirms the absence of high-molecular-weight nucleic acid contaminants in plasmid preparations isolated via acid-phenol extraction, even without the use of orthogonal purification methods (e.g., chromatography or filter cartridges). To confirm structural integrity, pMD2.G was digested with BamHI (lane 11) and BfuI (lane 12). Both yielded the expected fragments (Figure 2a), confirming that the plasmid was intact and that the >10 kb upper band in the uncut samples is a non-covalent dimer.

To rule out contamination with single-stranded DNA fragments, plasmid preparations were analyzed on overloaded agarose gels (5 µg per lane) stained with SYBR Green I (Figure 2b). No smear was observed in any of the overloaded lanes, confirming the absence of significant single-stranded DNA contamination.

### 3.3. Plasmid Yields and Purities Are Comparable Between Acid-Phenol and CsCl Methods

In our initial comparative experiments, the plasmids—the transfer vector pLV/CAR-GFP, the packaging helper psPAX2, and the envelope plasmid pMD2.G—were isolated from 50 mL bacterial cultures using each of the three purification methods.

Yields varied depending on the plasmid, with pLV/CAR-GFP specific yield: 0.89–0.99 mg/g, psPAX2 specific yield: 1.01–1.12 mg/g, and pMD2.G specific yield: 1.03–1.32 mg/g for the acidic phenol and CsCl gradient methods (Table 1). In contrast, the midiprep kit produced only 0.08–0.14 mg from the same culture volume (specific yields ranging from 0.30 to 0.38 mg/g depending on the plasmid)—approximately 3- to 4-fold lower than those of the manual methods. This substantial reduction in yield is consistent with the limited binding capacity of the anion-exchange column (approximately 100 µg, as stated by the manufacturer).

To determine whether the observed yield differences between the acidic phenol and CsCl methods were statistically significant, we performed Two-Way Repeated Measures ANOVA (RM ANOVA), with purification method (acidic phenol vs. CsCl) and plasmid type (pLV/CAR-GFP, psPAX2, pMD2.G) as within-subjects factors, and each plasmid-batch combination as the subject (six subjects: three plasmids × two batches). This design accounts for the fact that different plasmids inherently yield different amounts of DNA, irrespective of the purification method used. The analysis revealed no significant effect of purification method on plasmid yield (*p* = 0.0687). In contrast, plasmid type had a highly significant effect on yield (*p* = 0.0001), indicating that the inherent characteristics of each plasmid were the primary determinant of pDNA recovery.

The A_260_/A_280_ ratios obtained by CsCl gradient ultracentrifugation (1.81–1.92) were consistently higher than those from acidic phenol extraction (1.70–1.79) (Table 1), suggesting more efficient removal of protein and other UV-absorbing contaminants. To assess the statistical significance of this difference, we performed Two-Way Repeated Measures ANOVA on the purity data for these two methods, with the same design as above. This analysis revealed a significant effect of purification method (*p* < 0.0001), whereas plasmid type had no significant influence on purity (*p* = 0.8503).

Notably, the Qiagen Midi Kit gave the highest purity (A_260_/A_280_ = 1.89–1.94), but its yield was severely constrained by the column’s binding capacity, which limits its use to the manufacturer-recommended 50 mL culture volume.

### 3.4. Scale-Up and Endotoxin Measurements in Plasmid Preparations

Having established the baseline performance of the three methods using 50 mL cultures (Table 1), we evaluated the scalability of the acidic phenol and CsCl gradient methods by increasing the culture volume to 500 mL. Three independent replicate isolations were performed on different days with the manual methods. The commercial midiprep kit was not scaled up. However, we included the midiprep-purified plasmid batches from Table 1 as benchmarking controls in the endotoxin assays.

Plasmid batches were used to assess endotoxin levels using a chromogenic Limulus amebocyte lysate (LAL) assay. The yields and endotoxin levels from the scaled-up preparations are presented in Table 2.

The acidic phenol extraction method yielded plasmid preparations with endotoxin levels of 2.3–6.2 EU/µg pDNA—substantially lower than those obtained by single-round CsCl density gradient ultracentrifugation (14.6–31.2 EU/µg pDNA). The commercial midiprep kit, which incorporates a dedicated endotoxin removal step (Buffer ER followed by anion-exchange chromatography), produced plasmids with undetectable endotoxin levels (<0.1 EU/µg pDNA). Importantly, the endotoxin levels achieved by all three methods remained well below the threshold known to inhibit mammalian cell transfection (>2000 EU/µg DNA) [18]. These results confirm that the acidic phenol extraction removes endotoxin to levels compatible with efficient transfection and, notably, outperforms the single-round CsCl method in this regard.

The acidic phenol method demonstrated scalability under standard laboratory conditions, yielding 5–10 mg of plasmid DNA per 500 mL culture—sufficient to support lentiviral vector production in preparative amounts (>10^9^ TU), with potential for transition to industrial applications.

### 3.5. Functional Validation of Plasmids in Transfections for Lentiviral Vector Packaging

**Figure 3:**
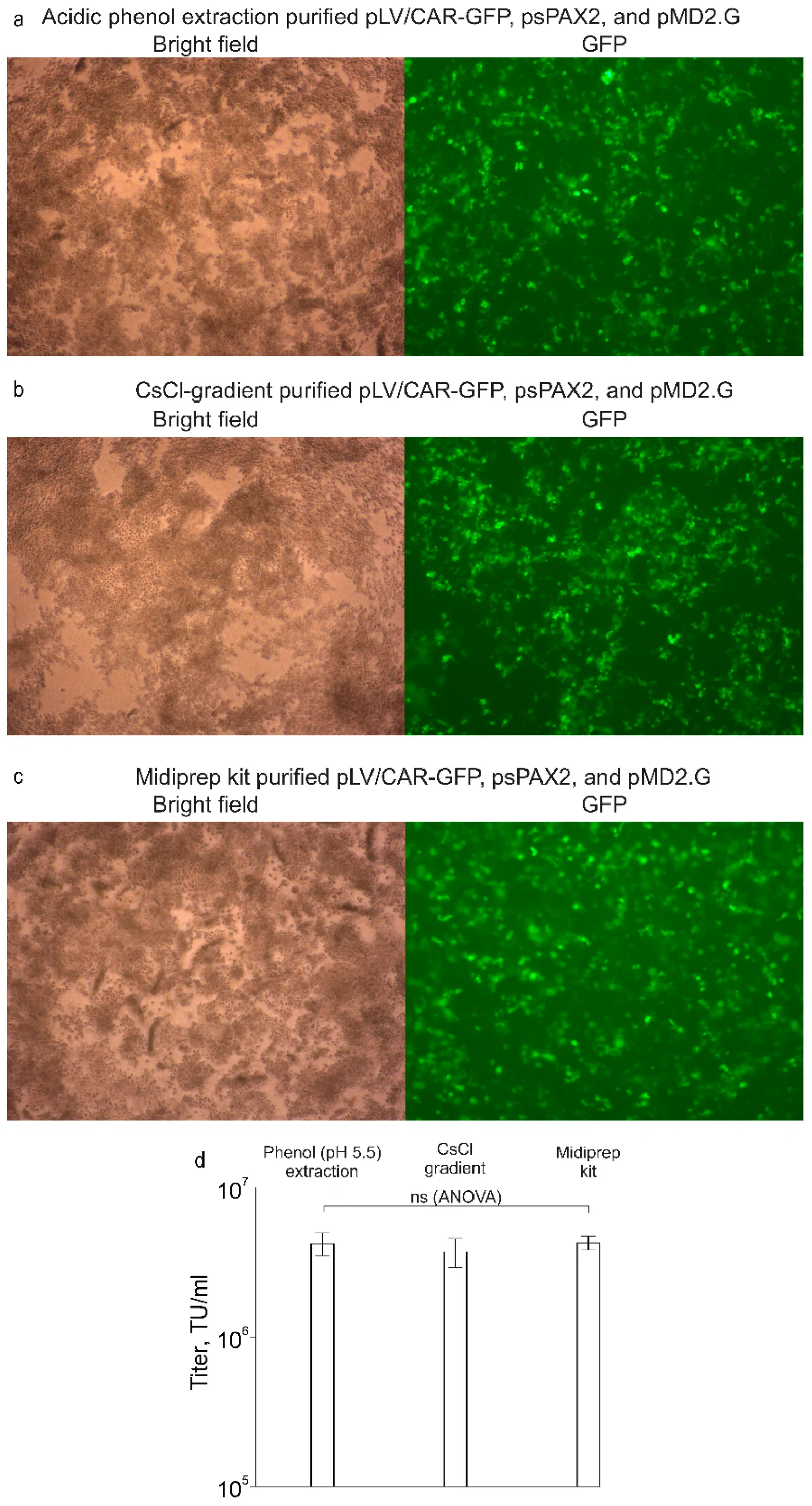
Plasmids purified by the acidic phenol extraction method perform as efficiently in lentiviral vector production transfections as those purified by CsCl density gradient ultracentrifugation or the commercial midiprep kit. HEK293FT cells were transfected with LV packaging mixtures (pLV/CAR-GFP, psPAX2, and pMD2.G) in which each plasmid was purified by the same method. (**a**–**c**) Representative phase-contrast (left) and GFP fluorescence (right) images at 48 h post-transfection for (**a**) the acidic phenol protocol, (**b**) the CsCl protocol, and (**c**) the Qiagen Midi Kit. 5× objective; scale bar: 100 μm. (**d**) Functional titers (TU/mL) in culture supernatants (mean ± SD, n = 3). One-way ANOVA detected no significant difference among the three groups (*p* > 0.05, n = 3).

To assess functional performance, we transfected HEK293FT cells with the scaled-up plasmid preparations obtained using the acidic phenol and CsCl methods (Table 2) and, for benchmarking, with midiprep-purified plasmids (Table 1). All three packaging plasmids were purified by the same protocol and used together for lentiviral vector production. Transfected cultures (Figure 3a–c) showed comparable morphology and GFP expression across all conditions.

Functional titers showed no significant difference among the three purification methods (*p* > 0.05), reaching ~5 × 10^6^ TU/mL (Figure 3d). Representative flow cytometry plots and GFP fluorescence histograms from titration experiments are provided in Supplementary Figure S1. Notably, the Qiagen Midi Kit-purified DNA, despite its superior purity, did not yield significantly higher titers than the other methods, confirming that the relatively simple manual methods provide pDNA of sufficient quality for efficient lentiviral vector production.

### 3.6. Comparison of Specific Activity of Lentiviral Vectors

To assess the quality of LVs produced with plasmids purified by the three methods, we measured the HIV-1 p24 capsid protein concentration in LV batches from the transfections described in Section 3.5 and computed the specific activity (TU/pg p24) (Table 3).

Specific activities were statistically indistinguishable (one-way ANOVA, n = 3) among the three purification methods, demonstrating functional equivalence of acidic phenol-purified plasmids to those obtained by the gold-standard CsCl method or commercial kit.

## 4. Discussion

The rapid expansion of cell and gene therapy (CGT) and nucleic acid vaccines has generated a pressing demand across academia and industry for high-quality plasmid DNA (pDNA), a critical raw material for lentiviral and AAV vector production [1,2]. This demand is particularly acute in CAR-T cell manufacturing—a rapidly expanding therapeutic modality—where the availability of transfection-grade pDNA for LV production remains a significant bottleneck [16].

In the field of pDNA production for CGT, industrial-scale manufacturing relies on sophisticated, multi-step chromatographic processes [3,19], whereas academic and emerging manufacturing facilities often depend on commercial kits that, despite their convenience, are costly and limited in scalability [9]. To address this gap, we developed an acidic phenol extraction method—a simpler, faster, and more cost-effective alternative to conventional approaches—with potential applications from small-scale to preparative amounts.

Our protocol is grounded in two well-established principles. The first is a modification of the alkaline lysis method described by Kado and Liu [20], which exploits the differential stability of DNA forms at high pH. Incubation of *E. coli* cells in 3% SDS at pH 12.6 selectively denatures linear chromosomal DNA, while covalently closed circular (ccc) plasmid DNA remains intact. Subsequent neutralization precipitates the denatured bacterial chromatin, leaving the plasmid in solution. Importantly, however, this original method was neither designed nor validated for purifying transfection-grade DNA.

The second, and more critical, foundation is the pH-dependent partitioning of DNA conformations between aqueous and organic phases, a principle detailed by Zasloff et al. [11] and refined by others [12,13]. At acidic pH (4.0–5.5), the reduced surface charge of relaxed or linear DNA forms (relative to highly supercoiled cccDNA) allows for their selective extraction into the organic phase while the plasmid DNA remains in the aqueous phase. We adapted this principle by performing extractions with buffered phenol to pH 5.5. Crucially, this same acidic environment protonates the phosphate groups of lipopolysaccharide (LPS), neutralizing its negative charge and promoting its efficient partitioning into the organic phase, a principle established in classical phenol-water extraction methods for endotoxins [21,22]. This dual functionality—simultaneously removing non-supercoiled DNA and endotoxin—is a key feature that enhances both the purity and the transfection suitability of the final pDNA preparation.

Our head-to-head comparison with the CsCl density gradient ultracentrifugation method, long considered the “gold standard” for pDNA isolation [10], and the widely used Qiagen Plasmid Midi Kit demonstrates the utility of our approach. The acidic phenol method achieved plasmid yields (5–10 mg per 500 mL culture) that were statistically equivalent to those of CsCl gradients and well within the expected range for high-copy-number plasmids [23]. While the Qiagen Midi Kit provided the highest purity (A_260_/A_280_ of 1.89–1.94), its binding capacity (approximately 100 µg per column) severely limits its scalability. In contrast, the acidic phenol extraction method is readily scalable; our preparations yielded sufficient plasmid DNA for both academic research and preparative laboratory production.

The slightly lower A_260_/A_280_ ratios observed for the acidic phenol method (1.70–1.79) compared with CsCl and kit controls may reflect trace residual phenol or co-extracted proteins. Nevertheless, plasmids purified by this protocol and stored in TE buffer (pH 7.2) have been used in our laboratory for over a year and have retained full functional activity in transfection and genetic engineering applications, as confirmed by preserved restriction patterns. While formal long-term stability studies have not yet been performed and are planned for the future, our existing practical experience indicates that residual impurities from the acidic phenol protocol do not adversely affect plasmid functionality under routine storage and handling.

The most critical validation was the functional performance of the purified plasmids. Cell morphology and GFP expression were comparable across all three methods, and functional lentiviral titers (~5 × 10^6^ TU/mL) and specific activities (40.88–43.99 TU/pg p24) were statistically indistinguishable, confirming that the purity achieved by the acidic phenol method is sufficient for efficient transfection and LV production. The endotoxin-free kit’s higher purity did not confer better functional performance, consistent with Butash et al. [9], who reported that transfection is inhibited only at endotoxin levels exceeding 2000 EU/µg DNA. We acknowledge that other bacterial components (e.g., CpG motifs, lipoproteins) may persist and potentially influence cellular responses; however, the functional equivalence of our preparations to controls indicates that any such contaminants do not compromise plasmid performance under the conditions tested.

During alkaline lysis, bacterial genomic DNA is denatured and fragmented, potentially generating single-stranded DNA fragments that may contaminate plasmid preparations if not efficiently removed. To evaluate this possibility, we analyzed plasmid preparations on agarose gels with intentional overloading (5 µg per lane) and staining with SYBR Green I, a dye with substantially higher sensitivity to single-stranded DNA compared with ethidium bromide. No smear was detected in either undigested plasmid DNA or restriction-digested samples (Figure 2b), confirming the absence of significant single-stranded DNA contamination in the final preparations.

Based on our extensive experience with this protocol, several practical considerations are worth noting. DNA pellets obtained in large-scale preparations may dissolve slowly; therefore, sufficient incubation with buffer on a shaker, with periodic checking, is recommended to ensure complete dissolution and avoid plasmid losses. Efficient organic extraction requires vigorous vortexing of the aqueous DNA solution with the organic solvent for at least 1 min to form a fine emulsion. This is followed by centrifugation at maximum speed for at least 5 min at room temperature to ensure the formation of a well-compacted interphase between the organic and aqueous layers. A dense interphase allows clean recovery of the aqueous phase without carrying over contaminants. Maintaining the room temperature of the tubes during centrifugation is important, as temperature shifts upon sample removal from the centrifuge may cause turbidity in the aqueous phase, which is resolved by re-centrifugation.

Beyond yield and functional performance, the acidic phenol method offers compelling practical advantages. It proved both faster and cheaper than the alternatives, requiring only standard laboratory equipment and avoiding the need for a costly infrastructure, such as ultracentrifuge or chromatography machine. Its main limitations are the handling of phenol, a toxic compound, and the slightly lower A_260_/A_280_ purity ratios. However, the latter does not appear to compromise downstream applications.

At laboratory scale, disposal of phenol- and chloroform-containing wastes is a routine practice, typically performed by authorized companies through incineration. At industrial scale, solvent recovery by distillation becomes economically attractive and reduces waste volumes. Scaling to industrial usage would necessitate closed extraction systems (e.g., batch extraction vessels with solvent recovery, as commonly employed in pharmaceutical manufacturing) and dedicated safety protocols. These engineering and waste-management measures are well established for hazardous organic solvents and do not constitute a fundamental barrier to further scale-up.

Other groups have explored alternatives to commercial kits, such as the Filterprep method [24] and magnetic bead-based approaches [25]. However, the scalability of these methods to the preparative scale (5–10 mg per preparation) is questionable, as they rely on adsorption matrices that scale proportionally with the amount of DNA. In contrast, our method is matrix-independent; its scalability is fundamentally limited only by the practical volumes that can be handled. This makes it uniquely suited to bridge the gap between small-scale research and the demands of vector production for early-phase clinical studies.

## 5. Conclusions

The acidic phenol extraction method is a robust, scalable, and cost-effective alternative to currently dominant methods, matching the yield and purity requirements for most applications. Residual endotoxin levels are negligible for downstream applications such as plasmid transfections for LV packaging. The method has become routine in our laboratory for producing plasmids used in both genetic engineering and CAR-T vector production.

## Figures and Tables

**Table 1:** Comparison of plasmid DNA yields and purity obtained by acidic phenol extraction, CsCl density gradient ultracentrifugation, and a plasmid midiprep kit.

Plasmid	Purification Method ^1^	Batch ^2^	Cell Pellet (Wet Weight, g)	Total Yield (mg)	Specific Yield (mg/g)	Purity (A_260_/A_280_)
pLV/CAR-GFP	CsCl	#1	0.27	0.266	0.99	1.86
		#2	0.27	0.240	0.89	1.81
pLV/CAR-GFP	Acidic phenol	#1	0.27	0.261	0.97	1.77
		#2	0.27	0.249	0.92	1.74
pLV/CAR-GFP	Midiprep	#1	0.27	0.081	0.30	1.89
		#2	0.27	0.092	0.34	1.91
psPAX2	CsCl	#1	0.31	0.313	1.01	1.92
		#2	0.31	0.322	1.04	1.89
psPAX2	Acidic phenol	#1	0.31	0.347	1.12	1.73
		#2	0.31	0.331	1,07	1.7
psPAX2	Midiprep	#1	0.31	0.109	0.35	1.94
		#2	0.31	0.117	0.38	1.93
pMD2.G	CsCl	#1	0.45	0.463	1.03	1.82
		#2	0.45	0.506	1.12	1.87
pMD2.G	Acidic phenol	#1	0.45	0.596	1.32	1.75
		#2	0.45	0.560	1.24	1.79
pMD2.G	Midiprep	#1	0.45	0.141	0.31	1.92
		#2	0.45	0.136	0.30	1.89

Comments: ^1^ For each plasmid, three purification methods are shown. Plasmid DNA was isolated from 50 mL cultures. ^2^ For each plasmid and purification method, two independent isolation experiments (batch #1 and batch #2) are shown.

**Table 2:** Yields and endotoxin levels in plasmid DNA preparations purified by three different methods.

Plasmid	Purification Method	Batch ^1^	Total Yield (mg) ^2^	Endotoxin (EU/µg pDNA) ^3^
pLV/CAR-GFP	CsCl gradient	#1	5.2	14.6
		#2	5.1	19.0
		#3	5.4	24.5
	Acidic phenol extraction	#1	5.4	2.3
		#2	5.8	3.4
		#3	5.5	5.4
	Midiprep kit	#1	—	<0.1
		#2	—	<0.1
psPAX2	CsCl gradient	#1	7.8	16.9
		#2	7.1	31.2
		#3	7.9	21.8
	Acidic phenol extraction	#1	8.1	4.3
		#2	8.0	3.6
		#3	7.7	3.3
	Midiprep kit	#1	—	<0.1
		#2	—	<0.1
pMD2.G	CsCl gradient	#1	7.4	14.8
		#2	7.8	26.4
		#3	7.9	18.2
	Acidic phenol extraction	#1	8.5	6.2
		#2	8.3	4.3
		#3	8.7	2.3
	Midiprep kit	#1	—	<0.1
		#2	—	<0.1

Comments: ^1^ Values are shown for three independent plasmid preparations (three batches per plasmid). ^2^ Isolations using the acidic phenol and CsCl methods were performed from 500 mL cultures. For the commercial kit, endotoxin measurements were performed on the preparations shown in Table 1. ^3^ Endotoxin levels were measured using the ToxinSensor Chromogenic LAL Endotoxin Assay Kit (GenScript, Piscataway, NJ, USA, Cat. No. L00350). Values below the limit of detection (0.01 EU/mL) are reported as <0.1 EU/µg pDNA.

**Table 3:** Comparison of lentiviral vector specific activity.

Plasmid Purification Method	Functional Titer (TU/mL) ^1^	p24 (ng/mL) ^1^	Specific Activity (TU/pg p24) ^1,2^
Acidic phenol extraction	4.24 ± 0.75 × 10^6^	103.3 ± 4.0	40.88 ± 5.66
CsCl gradient	3.75 ± 0.83 × 10^6^	90.0 ± 23.3	42.41 ± 6.98
Midiprep kit	4.29 ± 0.44 × 10^6^	98.0 ± 13.2	43.99 ± 3.33

Comments: ^1^ The data represent the mean ± SD of these three independent biological replicates. ^2^ No statistically significant differences were found between the methods (one-way ANOVA, *p* > 0.05). Data for individual LV batches are provided in Supplementary Table S1.

## Data Availability

Data is contained within the article or Supplementary Material.

## Supplementary Materials

The following supporting information can be downloaded at https://www.mdpi.com/article/10.3390/bioengineering13080939/s1. Figure S1. Representative flow cytometry histograms of GFP-positive cells from lentiviral titrations. HEK293FT cells (4 × 10^5^/well) were transduced with 200, 20, or 2 µL of viral supernatant and analyzed 48 h later. Panels (a–c) correspond to vectors produced with plasmids purified by acidic phenol extraction, CsCl gradient, and the midiprep kit, respectively. Numbers indicate the percentages of GFP^+^ cells used to compute the titers. Table S1. Functional titers, p24 concentrations, and specific activities of lentiviral vectors produced with plasmids purified by three different methods.

## Abbreviations

The following abbreviations are used in this manuscript:

AAV	Adeno-associated virus
CAR	Chimeric antigen receptor
CGT	Cell and gene therapy
CsCl	Cesium chloride
DMEM	Dulbecco’s Modified Eagle Medium
EU	Endotoxin unit
FBS	Fetal bovine serum
GFP	Green fluorescent protein
HIV	Human immunodeficiency virus
LAL	Limulus amebocyte lysate
LV	Lentiviral vector
p24	HIV-1 capsid protein
PBS	Phosphate-buffered saline
pDNA	Plasmid DNA
PEI	Polyethylenimine
RM ANOVA	Repeated measures two-way analysis of variance
SDS	Sodium dodecyl sulfate
SD	Standard deviation
TFF	Tangential flow filtration
TU	Transducing units
VSV-G	Vesicular stomatitis virus G protein
