# Supplementary material for: Scalable Production of Transfection-Grade Plasmid DNA by Liquid–Liquid Extraction Without Chromatography: Application to CAR-T Vector Packaging
Source: Bioengineering (Basel). 2026 Aug 20;13(8):939. doi: 10.3390/bioengineering13080939 (PMC13509930; doi:10.3390/bioengineering13080939)
Supplement: Supplementary file 1 [file bioengineering-13-00939-s001.zip › bioengineering-4501774-supplementary.pdf]

Supplementary material to

Scalable Production of Transfection-Grade Plasmid DNA by Liquid-Liquid Extraction Without Chromatography: Application to CAR-T Vector Packaging

Gaziza Nigmatulla, Aitolkyn Kydyrbayeva, Tolganay Kulatay, Gulzat Zauatbayeva, Bakytkali Ingirbay, Lyazzat Sagyndykova, Viktoriya Keyer, Dinara Zharlyganova, Maral Zhumabekova, Alexandr V. Shustov*

National Center for Biotechnology, Korgalzhin hwy 3/5, 010000, Astana, Kazakhstan

* Correspondence: shustov@biocenter.kz , Tel: +7-702-473-5305

G.N. Gaziza Nigmatulla	ORCID 0009-0001-7868-3396	nigmatullag@gmail.com
A.K. Aitolkyn Kydyrbayeva	ORCID 0009-0003-2199-5756	aitolkyn.yk@gmail.com
T.K. Tolganay Kulatay	ORCID 0009-0004-5885-8963	kulatay@biocenter.kz
G.Z. Gulzat Zauatbayeva	ORCID 0000-0003-1514-9302	zauatbaeva@biocenter.kz
B.I. Bakytkali Ingirbay	ORCID 0000-0002-6915-8207	ingirbay@biocenter.kz
L.S. Lyazzat Sagyndykova	ORCID 0009-0007-6561-0685	sagyndykova@biocenter.kz
V.K. Viktoriya Keyer	ORCID 0000-0001-8885-2387	keer@biocenter.kz
D.Z. Dinara Zharlyganova	ORCID 0000-0001-6383-4035	dinarazh@mail.ru
M.Z. Maral Zhumabekova	ORCID 0009-0002-9532-6232	zhumabekova@biocenter.kz
A.V.S. Alexandr V. Shustov	ORCID 0000-0001-9880-9382	shustov@biocenter.kz

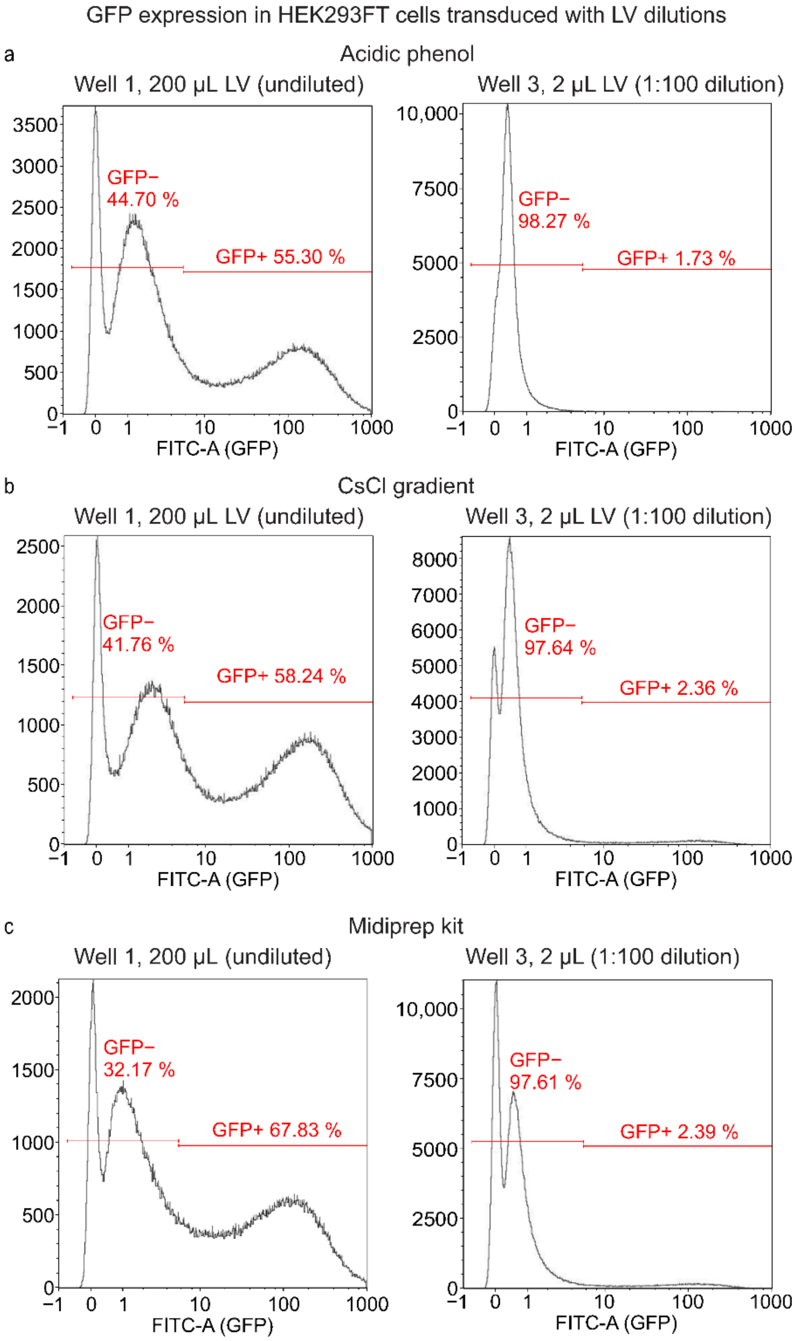

Figure S1. Representative flow cytometry histograms of GFP-positive cells from lentiviral titrations. HEK293FT cells (4×10^5/well) were transduced with 200, 20, or 2 µL of viral supernatant and analyzed 48 h later. Panels (**a**–**c**) correspond to vectors produced with plasmids purified by acidic phenol extraction, CsCl gradient, and the midiprep kit, respectively. Numbers indicate the percentages of GFP^+ cells used to compute the titers.

Table S1. Functional titers, p24 concentrations, and specific activities of lentiviral vectors produced with plasmids purified by three different methods.

Plasmid purification method [1]	Replicate [2]	Titer (TU/mL)	p24 (ng/mL)	Specific activity (TU/pg p24)
Acid phenol	1	3.45×10^6	99	34.85
Acid phenol	2	4.34×10^6	104	41.73
Acid phenol	3	4.93×10^6	107	46.07
CsCl gradient	1	3.33×10^6	94	35.43
CsCl gradient	2	4.71×10^6	111	42.43
CsCl gradient	3	3.21×10^6	65	49.38
Midiprep kit	1	3.92×10^6	83	47.23
Midiprep kit	2	4.77×10^6	108	44.17
Midiprep kit	3	4.18×10^6	103	40.58

Comments: [1] Plasmid DNA was isolated using three methods: acidic phenol extraction, CsCl density gradient ultracentrifugation, and the Qiagen EndoFree Plasmid Midi Kit, as described in the Materials and Methods section (Sections 2.4, 2.5, and 2.7). [2] Measurements are shown for three biological replicates for each plasmid purification method.
